# Thermal Plasticity of Multiple Traits Varies More Within Than Between Populations of *Plantago lanceolata* at Its Northern Range Edge

**DOI:** 10.1002/ece3.72201

**Published:** 2025-10-21

**Authors:** M. H. Hällfors, T. M. Robson, S. Burg, S. Pentikäinen, S. H. M. Koivusaari, M. Luoto, J. Nezval, R. Pech, M. Saastamoinen, L. E. Schulman, J. Sirén, H. Susi

**Affiliations:** ^1^ Organismal and Evolutionary Biology Research Programme, Faculty of Biological and Environmental Sciences University of Helsinki Helsinki Finland; ^2^ Finnish Environment Institute (Syke) Helsinki Finland; ^3^ UK National School of Forestry, University of Cumbria Cumbria UK; ^4^ Department of Biological and Environmental Science University of Jyväskylä Jyväskylä Finland; ^5^ Department of Geosciences and Geography University of Helsinki Helsinki Finland; ^6^ Botany and Mycology Unit, Finnish Museum of Natural History University of Helsinki Helsinki Finland; ^7^ Department of Physics, Faculty of Science University of Ostrava Ostrava Czech Republic; ^8^ Institute of Biotechnology FI‐00014 University of Helsinki Helsinki Finland

**Keywords:** common garden experiment, global climate change, inflorescence pigmentation, plant disease, ribwort plantain, virus

## Abstract

Temperature plays a pivotal role in defining the distribution of species and the fitness of individuals within species' ranges. Phenotypic plasticity can allow individuals to cope with varying environmental conditions, including rapid climate change. Populations at range edges experience more variable conditions than core populations and thus are hypothesized to exhibit higher thermal plasticity. However, as the strength of plasticity often varies between individuals, it can also differ among local populations at range edges. We studied the extent of and variation in thermal plasticity for several traits within and between populations of the perennial herb *Plantago lanceolata* L. (Plantaginaceae) at its northern range edge. We sampled seeds from nine sites within a 50 × 50 km region and grew them under three temperature regimes in a greenhouse. We measured traits related to size, flowering, pathogen responses, and inflorescence pigmentation. We expected to find higher plasticity in traits less strongly connected to fitness and that differences between individuals would outweigh differences between populations in underpinning this variation in plasticity. Our results show thermal plasticity in leaf size and abundance, flowering probability and abundance, and pigmentation. Notably, we also found increased pathogen symptoms and higher infection rates of one of two viruses screened, highlighting the potential for changes in pathogen sensitivity and exposure under climate change. Importantly, in all traits but flower abundance, more variation in plasticity was attributable to differences within populations than between populations. Although this contribution was small in magnitude compared to thermal effects on traits, the higher intra‐ versus interpopulation variation in plasticity suggests that differences between individuals provide most of the variation in thermal plasticity, which may be driven by small‐scale variations in habitat conditions, highlighting the need for conservation strategies that consider microhabitat variation to support short‐term adaptive responses to thermal variability.

## Graphical abstract

In this study, we investigate thermal phenotypic plasticity across multiple traits in *Plantago lanceolata* populations located at the species' northern range edge in the Åland Islands, Finland. Using a controlled greenhouse experiment with three temperature regimes, we assessed variation in vegetative, reproductive, pigmentation, and pathogen‐related traits across nine populations. Our findings reveal that while thermal plasticity is evident across several traits, the majority of variation in plasticity occurs within rather than between populations.

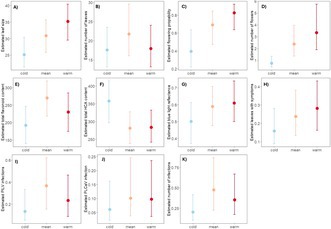

## 1 Introduction

Climate change is rapidly altering ecosystems, posing significant challenges to the survival and persistence of species (Pecl et al. 2017). The increasing frequency, intensity, and duration of extreme climatic events (Easterling et al. 2000; Rahmstorf and Coumou 2011; Rummukainen 2012) can have drastic effects on species by lowering individual fitness, causing direct breaches of thermal tolerances, decreasing competitive advantage, or lowering reproductive output (Kingsolver et al. 2013). Phenotypic plasticity entails that the phenotype is adjusted based on the prevailing environmental conditions. It can provide a crucial adaptive advantage to organisms under changing conditions (Bradshaw 1965). When conditions are variable, phenotypic plasticity is often the primary adaptive mechanism for many plant species (Matesanz et al. 2010; Sultan 1995) and can often help populations persist and adjust to new environmental conditions (Walter et al. 2023). However, not all genotypes or the populations that they comprise are equally plastic (de la Mata et al. 2022), and although not well‐studied, it can be expected that the relative extent of, and variation in, phenotypic plasticity within and between populations is likely to differ. Although not all phenotypic plasticity is adaptive (Ghalambor et al. 2007) and high plasticity can even can hinder long‐term adaptation (Oostra et al. 2018), acquiring a better understanding of plasticity patterns for different traits would help us understand the adaptive capacity versus vulnerability of populations exposed to climate change and further help guide management strategies that promote species persistence in a changing climate.

Populations from the high latitude edge of the range of a species often exhibit stronger plasticity overall, but less local adaptation in their mean and plasticity of trait values, compared to populations at the core of the range (Ghalambor et al. 2007; Rehm et al. 2015) because these environments often vary greatly both within and between years (Leung et al. 2020). Thermal plasticity, in particular, can be positively correlated with increasing latitude and is likely adaptive in high‐latitude populations where the growing season is short and relatively cool, but thermal variation is high (Marshall et al. 2019). Simultaneously, populations at the leading range edge are at the forefront of potential range shifts induced by climate change, as they are the most likely populations from which genotypes would colonise new suitable areas outside of the current range (Matesanz et al. 2010; Rehm et al. 2015; Walter et al. 2023). If such populations also have high variation in thermal plasticity, this can help them persist under climate change by providing sufficient standing variation in plasticity to support the evolution of novel response norms, overcoming intermittent suboptimal conditions, and colonising newly suitable areas (Brancalion et al. 2018; Hendry 2016; Walter et al. 2023).

Variation in plasticity can be measured at both the intra‐ and interpopulation level (Murren et al. 2015) and for several different traits. Traits closely tied to fitness are expected to undergo more intense selection, resulting in lower plasticity (Bradshaw 1965; Stearns and Kawecki 1994; Villellas et al. 2021), while traits less directly linked to fitness are likely to exhibit higher plasticity, allowing individuals to adjust to short‐term environmental changes (Sultan 1995; Villellas et al. 2021). Among populations, the mean thermal plasticity of any trait may vary (1) if habitat and thermal conditions within the sites of the local populations tend to vary to a different degree than the average variation between sites, and (2) if gene flow between populations is not substantial enough to dilute local adaptation in plasticity in the short term. Such variation in the plasticity of fitness‐related traits can increase the potential for genetic rescue and the evolution of responses suitable for novel conditions in the long term (Aspinwall et al. 2015; Droste et al. 2010; Sgrò et al. 2011). Within populations, individuals may vary in their thermal plasticity, for example, if the habitat contains a wide range of microenvironments that cause heterogeneity in thermal conditions. Such intrapopulation variation in thermal responses can provide a buffer against the impact of climate change by allowing some individuals to survive during suboptimal years and quickly begin repopulating a site with their offspring, without necessarily requiring recolonization from surrounding populations.

**FIGURE 1:**
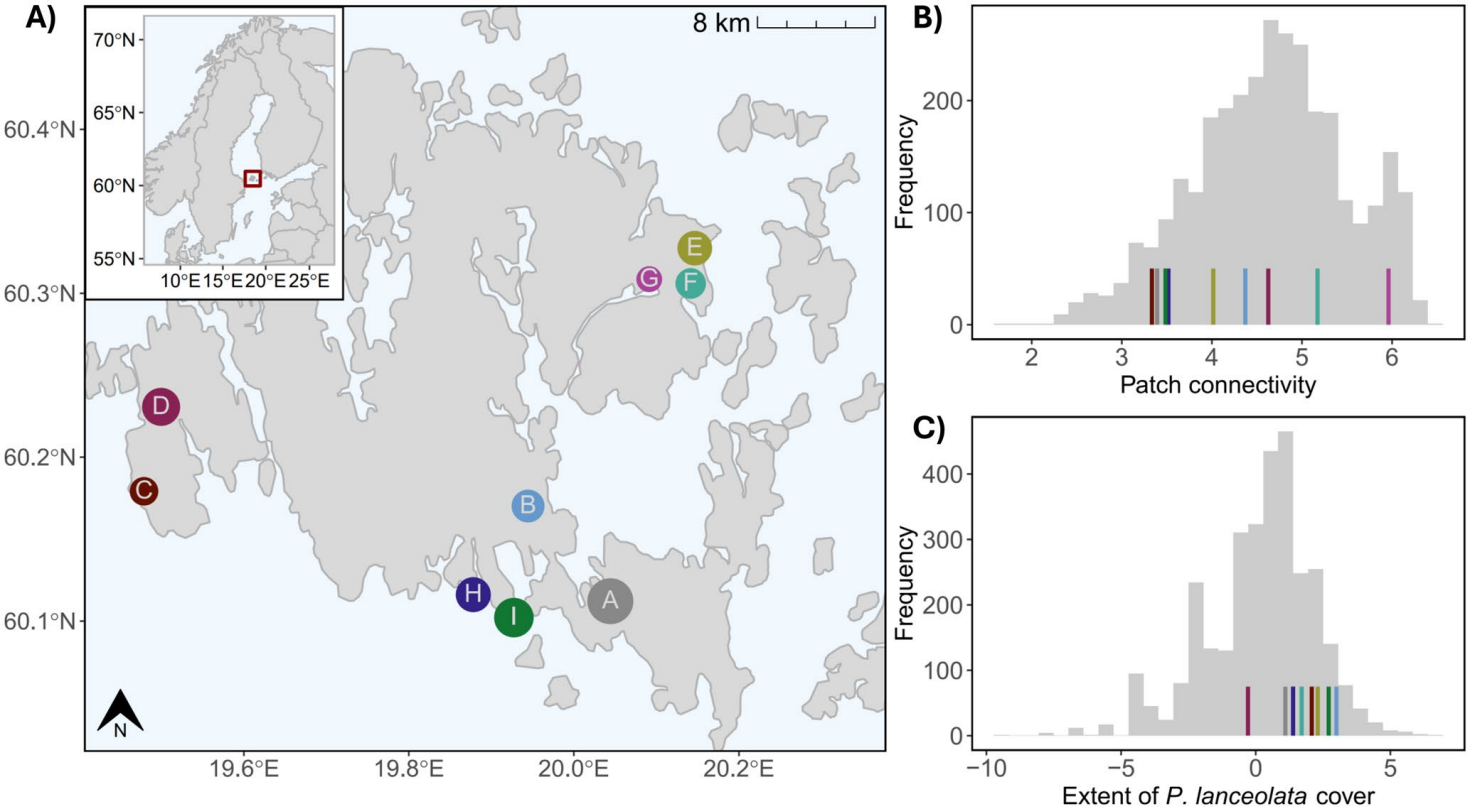
Seed collection sites in Åland. (A) Seed collection sites marked by colours and letters, point size represents the relative size of each habitat area, i.e., the area corresponding to an annually inventoried patch in the *Melitaea cinxia* metapopulation system (Ojanen et al. 2013), ranging from 1079 m^2^ in sampling site G to 8661 m^2^ in sampling site A. Inset shows Northern Europe with the Åland Islands indicated by a red box. Distribution of (B) patch connectivity and (C) the extent of *P. lanceolata* cover in 2019 across the Åland *Melitaea cinxia* metapopulation system, where coloured lines indicate the values for each of the seed collection sites (colours as in A).

We studied multi‐trait variation in thermal phenotypic plasticity within and between nine populations of the widespread perennial herb *Plantago lanceolata* L. (Plantaginaceae) sampled within a 50 × 50 km region at its high‐latitude range edge in the Åland Islands, Finland (Figure 1). *P. lanceolata* is not a threatened species; it has a wide geographical range and occurs across a broad set of habitats and climates. However, as the abundance of *P. lanceolata* within its sites of occurrence is vital to support many species sharing the same habitat patches (Opedal et al. 2020), fluctuations in its local abundance can have large effects on dependent species, underscoring the need to understand its dynamics in the context of climate change. Using a greenhouse experiment to create three temperature treatments that represent the range of conditions that the populations may currently experience in this region, we (1) estimated the extent of thermal plasticity for a suite of traits, ranging from vegetative and flowering traits to pathogen responses and virus infections, and (2) tested whether most of the variation in thermal plasticity lies at the intra‐ or interpopulation level.

The studied populations originate at high latitudes, leading us to expect to find strong plasticity to temperature. Previous studies of *P. lanceolata* have found a positive latitudinal gradient for increasing phenotypic plasticity of inflorescence spike reflectance and color (Lacey et al. 2010; Lacey and Herr 2005; Marshall et al. 2019)—traits which likely act to thermoregulate the inflorescences. Across its wide native distribution through Eurasia, this species exhibits high phenotypic plasticity especially for traits that have relatively little connection to fitness (such as biomass; Villellas et al. 2021). Accordingly, we expected to find higher plasticity in such traits compared to those, such as flowering‐related traits, more strongly connected to fitness. We also tested whether the intrapopulational variation in phenotypic plasticity is higher than the average variation in plasticity between populations. As microclimatic conditions within the local habitats where the species occurs in Åland likely vary substantially, we assumed this would have resulted in high variation in plasticity within populations. In addition, while the mean temperature in Åland has increased during the past decades (Figure S1), weather conditions have recently also become more synchronous across this region (Kahilainen et al. 2018; van Bergen et al. 2020), which may lately have reduced variability in plasticity between local populations. Thus, we expected to, overall, find more variation in plasticity within populations than between populations.

## 2 Materials and Methods

### 2.1 Plant Material

We collected seeds of *P. lanceolata* from nine local populations (habitat patches consisting of meadows or pastures) in Åland during August 2019 (Figure 1; Table S1). In the Åland Islands, *P. lanceolata* occurs in a network of ca. 4000 meadows (Ojanen et al. 2013). We chose the seed collection sites for this study from nine populations in three different parts of mainland Åland to represent (1) a variety of areas across the Åland main island, (2) *P. lanceolata* habitat area extent, and (3) connectivity to other nearby local populations of *P. lanceolata* (Figure 1).

All meadows in Åland where *P. lanceolata* occurs are annually surveyed for the presence of the butterfly species *Melitaea cinxia*, the larvae of which use it as their food plant, and for the fungal pathogen *Podosphaera plantaginis* that infects *P. lanceolata* (Ojanen et al. 2013). During the first surveys in the beginning of the 1990s, the cover of *P. lanceolata* and *Veronica spicata* (the other host species of *M. cinxia* larvae) was also measured and their exact locations recorded (Ojanen et al. 2013). As a proxy for *P. lanceolata* population size, ground cover (m^2^) of the plant has been estimated within each patch (Ojanen et al. 2013). Habitat area is considered the extent to which the two host plant species of *M. cinxia* occur in a patch. This measure of habitat area can be used as a proxy for fragmentation and population size and thus a rough proxy for genetic diversity (González et al. 2020). Connectivity between patches is evaluated annually, and can be used as a proxy for gene flow among populations (Hanski 1999). *Plantago lanceolata* population connectivity was calculated as:

$$S_{i}^{L}=\sum \exp^{-{\mathrm{\alpha d}}_{\mathrm{ij}}}\sqrt{A_{j}}.$$

where *d*_*ij*_ is the Euclidian distance between patches *j* and *i*, and *α* is the parameter of the negative exponential dispersal kernel, which was set to 1 km^−1^ (see Jousimo et al. 2014 for more details). *A*_*j*_ is the area (m^2^) of habitat patch *j*.

As *P. lanceolata* was the most common or the only host species in the seed collection sites for this study (Ojanen et al. 2013), habitat area corresponds well with the extent of the focal species. Based on the connectivity metrics and the species' dispersal ability (with both pollination and seed dispersal mainly occurring within local populations), we consider each sampling site to be independent from the others and treat them as spatially unstructured variables in our subsequent analyses. However, to test that closely located sites with high connectivity values within regions are not more similar to each other than expected, we included a sensitivity analysis testing for consistency of results by leaving out one of the sites within the region in turn (see Section 2.4 below).

To obtain a representative sample of the diversity within each population, we collected seeds from 17 to 50 individuals in each of the nine chosen sites (i.e., per population; individuals sampled based on availability, following seed collecting guidelines; ENSCONET 2009; Table S1). We collected seeds from each mother individual separately to allow the seeds of different mother plants to be sown across all treatments to measure within‐population variation in plasticity. We deposited voucher specimens from each sampling site at the herbarium of the Finnish Museum of Natural History (H *sensu* Thiers 2016). We left the seeds to dry and ripen at room temperature for a few weeks before the start of the experiment.

### 2.2 Experimental Set‐Up and Thermal Conditions

We conducted the experiment in the Viikki Plant Growth Facilities at the University of Helsinki during September 2019–February 2020, in six greenhouse compartments with three temperature treatments each replicated once. The temperatures chosen for the treatments were based on average June and July temperatures in Åland, 1959–2018 (Figure S1). The Cold treatment day temperature was set at 17°C with a night temperature of 8°C, while the Mean temperature treatment was 20°C during the daytime and 11°C at night, and the Warm treatment was set at 23°C during the daytime and 14°C at night. High pressure sodium lamps created light conditions and photoperiod consistent across all treatments (18 h light and 6 h dark), with day and night temperatures following the photoperiod.

From each of the nine sampled populations, we randomly chose the seed lots of 12 mother individuals. From each of these seed lots, we randomly chose 12 seeds for the experiment (a total of 1296 seeds). We assumed that this would be sufficient to result in at least 6 germinated seedlings from at least 10 mother individuals (one for each replicate, two per treatment; totaling 540 plants for the experiment). Seeds were sown in mid‐September 2019 during 4 consecutive days. One month after sowing, we chose the experimental individuals by stratified random sampling from the entire pool of available germinated seedlings. Altogether, 517 plants were included in the experiment, with 82–92 individuals in each replicate (90 and 92 in the cold replicates; 85 and 86 in the mean replicates; and 82 in both warm replicates). See the more detailed description on experimental set‐up and plant treatments in Dryad.

### 2.3 Trait Measurements

To test thermal plasticity, we measured vegetative traits (leaf size and number), floral traits (flowering probability and abundance), floral bract pigmentation—content of both flavonoids and hydroxycinnamic acid (HCA) derivatives (including phenylethanoids), and floral blue‐light reflection as a proxy of pigmentation, and the proportion of leaves showing symptoms of pathogen infection. We also tested for the presence of two common viruses: *P. lanceolata* latent virus (PlLV; Susi et al. 2017) and *Plantago* latent caulimovirus (PLCaV) and co‐infections by the two viruses (Susi et al. 2019). See the more detailed methods description in Dryad for motivation for choosing to measure these traits. The measured trait values across populations are given in Figure S4 and separately for each population in Figure S5. All data are available in Dryad.

We took leaf size measurements during three consecutive days starting 45 days since sowing began. We measured the length and width in cm of the leaf that, based on visual inspection, appeared to be the longest. We counted the number of leaves on each individual plant 12 weeks from sowing. Simultaneously, we assessed the degree to which plants showed symptoms of vertical pathogen infection (pathogens that likely had arrived with the seeds) by separately counting the number of leaves with typical signs of pathogen infection symptoms. We noted the number of leaves showing different kinds of pathogen symptoms (red, yellow, curly, necrosis, undefinable). We used the proportion of leaves showing any symptoms as pathogen response in our analyses. The information on different categories of symptom is available in the accompanying data in Dryad. To measure infection rate by two common pathogens, we also took leaf samples from each individual for virus detection by cutting a 1 cm^2^ piece from a leaf of each individual and placing the sample in a micro‐tube. We froze and kept the samples at −20°C until DNA extraction. We extracted the DNA following Lodhi et al. (1994). See method description in Dryad for information on how viruses PlLV and PlCaV were detected. The pathogen responses thus measure how the load of pathogens introduced in the experiment through the seeds affects infection rate and symptoms in plants grown at different temperatures. While these measurements allow us to capture the outcome of pathogen effects in the different thermal treatments, this does not allow us to distinguish any differences in resistance among the plants themselves or whether the pathogens differed in their virulence across the different temperatures tested.

19 weeks into the experiment, we began collecting inflorescences. We collected only fresh mature inflorescences, just before stigma, petals, and anthers emerged from between the sepals. We collected inflorescences 1–3 times per week, based on their availability. By week 22, we had collected inflorescences from 72, 155, and 130 individuals in the Cold, Mean, and Warm treatments, respectively. These were used to measure floral bract pigmentation (flavonoids and HCA derivative content) and floral blue light reflection (see method description in Dryad for information on how this was conducted). For those individuals from which several inflorescences were collected, we averaged all subsequent measurements per individual prior to statistical analyses. After inflorescence collection for reflectance and pigmentation analyses was completed, we counted the total number of flowers by tallying the remaining inflorescences and previously cut flowering stalks. We summarized flower presence by truncating flower numbers > 1 to 1.

### 2.4 Statistical Analyses

Our main aim was to assess, for the different traits, (1) the degree of thermal plasticity and (2) the origin of the majority of the variation in this plasticity, i.e., whether most variation in plasticity could be assigned to the population level or to the mother individual level. A mixed modeling approach allowed questions (1) and (2) to be assessed simultaneously, by choosing *temperature treatment* as a fixed effect and *population* and *mother individual* as nested random effects (Arnold et al. 2019). Here, the fixed effect describes the overall average response to the temperature treatment of the focal population, while the random effects describe how much variation is partitioned among *populations* and *mother individuals*, i.e., the contribution of variation in response attributable to interpopulation and intrapopulation differences, respectively. Using this approach, we were able to answer the main research questions concerning the contribution to variation in the measured traits by different local populations and mother individuals as opposed to the average value of all populations. Simultaneously, we accounted for non‐independence between plants grown in the same greenhouse chamber, by also using greenhouse chamber as a random effect (*Replicate*). We evaluated the independence of the populations within each of the three main sampling areas by their connectivity values (Figure 1). For populations with relatively high connectivity values and situated in close proximity to each other (the region containing populations G, E, and F), we conducted a sensitivity analysis to test the consistency of the results. We left out one of the highly connected populations at a time and compared the model estimates, posterior probabilities, and SDs.

We used the *brms* package (version 2.15.0; Bürkner 2021) in the R environment (R version 4.2.2; R Core Team 2024) to fit generalized linear mixed‐effect models (GLMMs and LMMs) in a Bayesian framework using several different error distributions. We modeled the continuous traits *leaf size, total flavonoid content, HCA content*, and *blue light reflectance* with a Gaussian error distribution. We estimated leaf size as the product of leaf length and leaf width to obtain a rough estimate in cm^2^. These values were divided by 100 and square‐root transformed, providing a fit for the Gaussian error distribution.

The count variable *number of leaves* showed overdispersion, and we modeled it with a negative binomial distribution. The other count variables, *number of flowers* and *number of infections*, were modeled with a zero‐inflated Poisson distribution to account for their high numbers of zeros. The binary traits, *flowering*, *PLCaV infection*, and *PlLV infection*, were modeled with a Bernoulli error distribution. Finally, we modeled the *proportion of leaves with pathogen responses* with a binomial error distribution on the numbers giving rise to the proportion.

For all models, we used informative prior distributions, which were needed to avoid computational issues. The informative prior distributions were justified for use here because the magnitude of the effects was expected to be small for all response variables. For the fixed effects and for the random effects, we used Gaussian distributions with a mean of zero for both and a SD of 2 and 1, respectively. However, for the models on the HCA derivative and flavonoid content, we set the SD to a 100 for the fixed effects and 50 for the random effects to account for the larger scale of these response variables. For the intercept, we used Student's t as defined by the default settings in *brms* (Bürkner 2021). For all models, we ran four chains with 4000 iterations including a warm‐up of 2000 iterations and a thinning rate of 1. Thus, we obtained 8000 posterior samples for each model.

For error distributions with an additional parameter to the location parameter, we considered a version of the model where the additional parameter was allowed to vary by treatment temperature to account for distributional differences between temperature treatments. For response variables with a Gaussian error distribution (*leaf size*, *flavonoid*, and *HCA content*, *blue light reflectance*), the additional parameter was the *residual SD* (*σ*). For the *number of flowers* and *number of infections* modeled with a zero‐inflated Poisson distribution, the additional parameter was the *zero‐inflation probability*. For the *number of leaves*, modeled using a negative binomial distribution, the additional parameter allowed to vary was *shape*. We compared the basic models to those models including this varying error distribution using the *loo_compare* function within the *brms* package, which compares the predictive performance of the models with leave‐one‐out cross‐validation (Vehtari et al. 2017). Because of computational issues, the comparison of the number of leaves was conducted using the widely applicable information criterion (waic; Vehtari et al. 2017). If the additional model did not perform better (uncertainty intervals overlapped), we used the basic model for interpretation and result presentation.

We evaluated model convergence by investigating whether the Rhat values were < 1.05 and the bulk effective sample size and tail effective sample size were each > 400. We also visually inspected plots of posterior predictive checks using 10 posterior samples to identify potential discrepancies between the observed and predicted data.

In addition to interpreting the results based on mean posterior probability distribution and credible intervals, we used the *hypothesis* function in the *brms* package to interpret the models vis‐à‐vis the effect of temperature treatment. This function computes an evidence ratio for a one‐sided hypothesis. In other words, we asked what the posterior probability is of the response being bigger (or in the case of phenolic absorbance where the direction of the effect was negative across treatments, smaller) than zero: (a) for the mean temperature, (b) between the mean and the warm temperature, and (c) in the warm temperature.

We did a correlation analysis using *ggpairs* function from *GGally* package (Schloerke et al. 2024) on all measured traits, both across and within treatments, to evaluate the degree to which, e.g., high values in certain traits correlate with high values in others.

To estimate the effect on variance in thermal plasticity resulting from intrapopulation and interpopulation differences, we compared the estimated SD attributable to the random effect of population and mother individual.

## 3 Results

### 3.1 Thermal Plasticity in the Measured Traits

**FIGURE 2:**
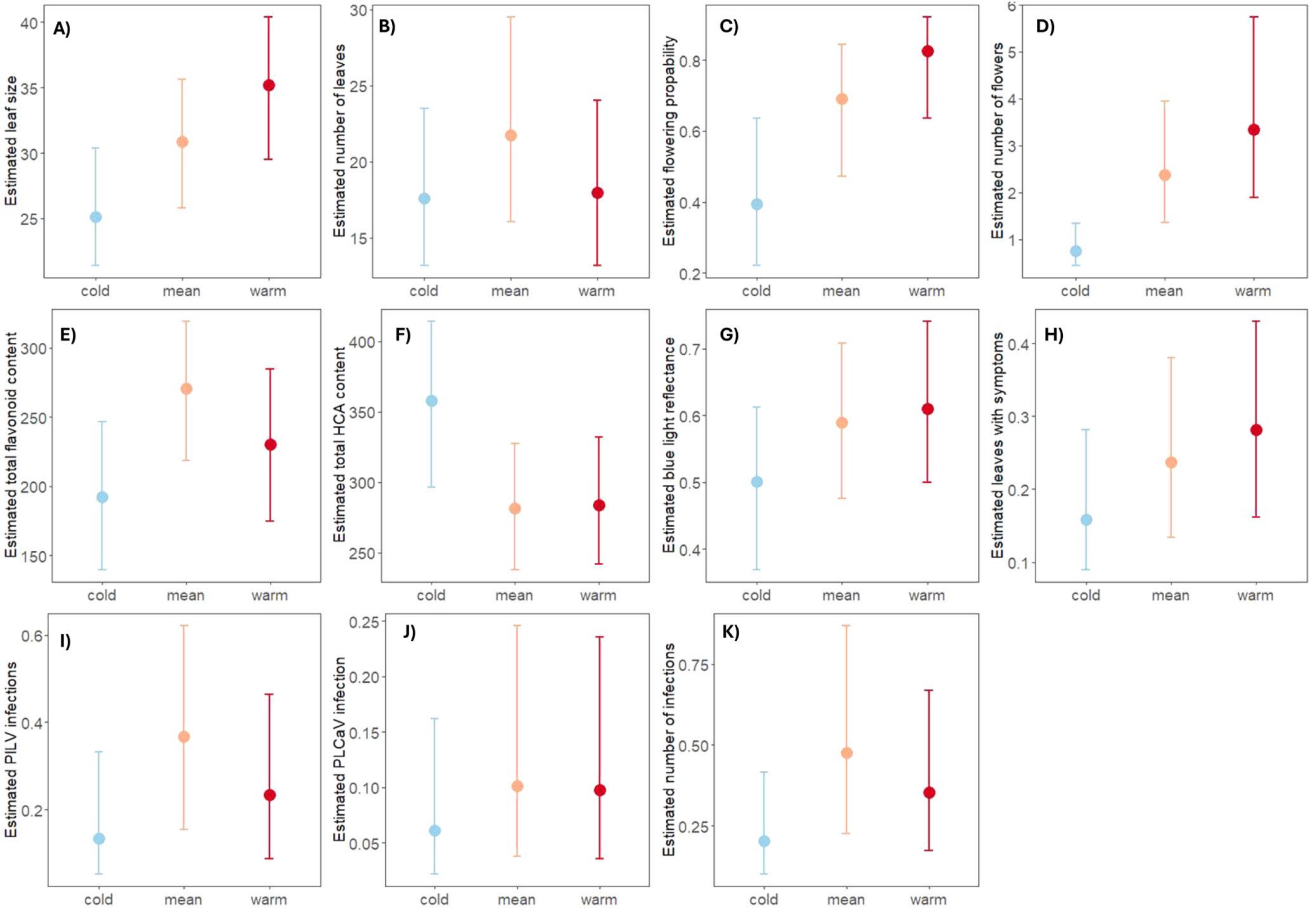
Posterior mean and credible intervals for the average response per treatment temperature. (A) Leaf size, (B) number of leaves; (C) flowering probability; (D) number of flowers, E) flavonoid content; (F) HCA content; (G) blue light reflection; (H) proportion of leaves with pathogen symptoms; (I) probability of PLCaV infection, (J) probability of PlLV infection, (K) probability of virus infections. Estimated effects (on the latent, i.e., modelled, scale) in Table 1. See Figure S7 for corresponding results per population.

We found moderate to strong evidence (following the evidence language suggested by Muff et al. 2021; Table 1) that plants in the mean and warm thermal treatments had bigger leaves (posterior probability of the response [hereafter PPR] between the mean and the warm treatment > 0 = 0.96 and 0.99, respectively), flowered more often (PPR = 0.97 and 0.99, respectively), and had more flowers (PPR = 0.98 and 0.99, respectively) than those in the cold treatment (Table 1; Figure 2; Figure S7 for estimated responses per population). We found weak evidence that individuals had bigger leaves and flowered more often in the warm than in the mean thermal treatment (PPR = 0.93 for both leaf size and flowering probability) and for producing more leaves in the mean compared to the cold (PPR = 0.91).

**TABLE 1:** Summary of model statistics per response. Mean of the posterior probability distribution with lower and upper credible intervals and evidence ratio for a one‐sided hypothesis. Estimates are on the linear predictor scale. See Figure 2 for estimates on the scale of the data. The evidence ratio for a one‐sided hypothesis asks what the posterior probability is of the response being bigger (or in the case of total HCA content where the direction of the effect was negative across treatments, smaller) than 0 in the mean temperature, in the warm temperature, and between the mean and the warm temperature. For interpreting the posterior probabilities, we followed the evidence language suggested by Muff et al. (2021), interpreting values of 0.9–0.94 as week evidence, 0.95–0.98 as moderate evidence and values above 0.99 as strong evidence. The posterior probability values in the three rightmost columns are marked in bold when they exceed the weak evidence threshold of 0.9.

Response variable group	Response variable	Estimate in cold (±CI)	Estimate in mean (±CI)	Estimate in warm (±CI)	Post. prob. of the resp. in the mean > 0	Post. prob. of the resp. in the warm > 0	Post. prob. of the diff. btw warm and mean > 0
Leaf and flowering responses	Leaf size (√cm^2^)	5.0 (4.6, 5.5)	5.5 (5.1, 6.0)	5.9 (5.4, 6.4)	**0.96**	**0.99**	**0.93**
	Number of leaves	2.9 (2.6, 3.2)	3.1 (2.8, 3.4)	2.9 (2.6, 3.2)	**0.91**	0.1	0.57
	Flowering probability	−0.4 (−1.3, 0.6)	0.8 (−0.1,1.7)	1.6 (0.6, 2.5)	**0.97**	**0.99**	**0.93**
	Number of flowers	0.3 (−0.2, 0.9)	1.2 (0.7, 1.7)	1.4 (0.8, 1.9)	**0.98**	**0.99**	0.8
Pigmentation responses	Total flavonoid content	192.5 (139.7, 246.4)	270.2 (218.4, 318.9)	229.8 (174.9, 284.5)	**0.98**	0.08	0.86
	Total HCA content	361.3 (307.7, 411.2)	282.9 (236.3, 332.3)	284.8 (235.3, 337.1)	0.02	0.53	0.02
	Blue light reflectance	0.5 (0.4, 0.6)	0.6 (0.5, 0.7)	0.6 (0.5, 0.7)	**0.93**	**0.95**	0.64
Pathogen responses	Leaves with symptoms	−1.7 (−2.3, −0.9)	−1.2 (1.9, 0.5)	−0.9 (−1.7, −0.2)	0.89	**0.95**	0.75
	PlLV infections	−1.8 (−2.9, −0.7)	−0.6 (−1.7, 0.5)	−1.2 (−2.3, −0.1)	**0.95**	0.16	0.85
	PLCaV infection	−2.7 (−3.8, −1.7)	−2.2 (−3.2, −1.1)	−1.2 (−2.3, −0.1)	0.82	0.47	0.80
	Number of infections	−1.5 (−2.2, −0.7)	−0.7 (−1.5, −0.1)	−1.0 (−1.7, −0.3)	**0.94**	0.21	0.86

We found moderate support for differences in flavonoid content being higher in the mean compared to the cold (PPR = 0.98). We also found weak to moderate evidence for greater blue reflectance between the mean and warm thermal treatments compared to in the cold (PPR = 0.93 and 0.95, respectively). There was no support for differences in HCA content between the treatments.

Finally, concerning pathogen responses, we found moderate support for there being more symptoms in the warm treatment compared to in the cold and more PlLV infections in the mean than in the cold thermal conditions (PPR = 0.95 for both). We found weak evidence of more infections in the mean compared to the cold (PPR = 0.94). There was no support for differences in Caulimo virus infections between the treatments (Table 1; Figure 2; Figure S7 for estimated responses per population).

When the additional distributional parameter was allowed to vary between temperature treatments, the model fits were improved for all models except those for the number of leaves, flavonoid content, and HCA content (Table 2). This indicates that for leaf size, number of flowers and infections, and blue light reflection, variation in the response differed between temperature treatments. For leaf size, the individual variation was largest in the warm treatment, whereas for blue‐light reflection, individual variation was largest in the mean temperature treatment. For the number of flowers and infections, the probability of zero inflation in the data was largest in the cold temperature treatment.

**TABLE 2:** Error distributions for models with an additional parameter to the location parameter. For some of the models, we considered a version of the model where the additional parameter was allowed to vary by treatment temperature. ELPD difference (SE difference) indicates the difference between the basic model and the model where additional parameters are allowed to vary by treatment. When the model version including this parameter did not explain the data better, we do not present estimates of the additional parameter per treatment.

Response variable group	Response variable	Additional parameter	ELPD difference	Estimate of the additional parameter (lower CI, upper CI) in the treatments		
				Cold	Mean	Warm
Leaf and flowering responses	Leaf size (√cm^2^)	Residual SD *σ*	−18.5 (9.2)	0.6 (0.6, 0.7)	0.6 (0.5, 0.7)	0.9 (0.8, 1.1)
	Number of leaves	Shape	−1.1 (7.0)	NA	NA	NA
	Number of flowers	Zero‐inflation prob.	−5.2 (3.8)	0.5 (0.3, 0.6)	0.3 (0.2, 0.4)	0.2 (0.1, 0.3)
Pathogen and pigmentation responses	Number of infections	Zero‐inflation prob.	−0.3 (0.2)	0.02 (0.0, 0.4)	0.003 (0.0, 0.1)	0.004 (0.0, 0.2)
	Total flavonoid content	Residual SD *σ*	0.0 (1.7)	NA	NA	NA
	Total HCA content	Residual SD *σ*	−3.0 (5.6)	NA	NA	NA
	Blue light reflectance	Residual SD *σ*	−4.5 (3.1)	0.1 (0.1, 0.1)	0.1 (0.1, 0.1)	0.2 (0.1, 0.2)

The measured trait types showed low correlation among each other (Figure S6), both across and within treatments. This indicates that the same individuals did not tend to produce high or low trait values for measured traits of different categories (vegetative, flowering, inflorescence pigmentation, pathogen responses).

### 3.2 Intrapopulational Versus Interpopulational Variation in Plasticity

**FIGURE 3:**
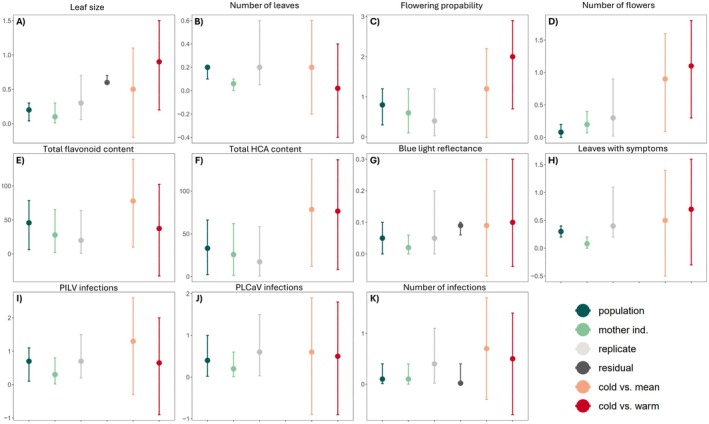
Visualisation of the mean impact and credible intervals of random effects, residuals, and fixed effects. (A) Leaf size, (B) number of leaves; (C) flowering probability; (D) number of flowers, (E) flavonoid content; (F) HCA content; (G) blue light reflection; (H) proportion of leaves with pathogen symptoms; (I) probability of PLCaV infection; (J) probability of PlLV infection; (K) probability of virus infections. The impact measures are ordered from right to left on the *x*‐axis as follows: The random effects population (dark green), mother individual (light green) and replicate (light grey), residual effects (dark grey; presented only for models where this parameter is well defined), and fixed effects difference between mean and cold (peach) and between warm and cold (red). The points show the mean impact and the line ranges show their credible intervals. The impact of random and residual effects are SDs attributable to them while the impact of the fixed effects are the estimated differences in trait values in the mean and warm temperatures, respectively, compared to the cold. For total HCA content where the direction of the fixed effect was negative across treatments, the estimates for fixed effects were converted to absolute numbers, to allow comparison to SD of random and residual effects.

The estimated SD attributable to the random effects *population* and *mother individual* were low for all response variables (Table 3; Figure 3; Figure S5 shows estimated responses per population) compared to the SD attributable to *replicate* (except for flowering probability and number of infections where the SD attributable to *replicate* was smaller than or equal to, respectively, the variation attributable to *population* or *mother individual*). For leaf size and blue light reflectance, but not for number of infections (models for which the residual SD is well defined), the SD attributable to population was smaller than the residual SD. Also, compared to the estimated differences between treatment temperatures (fixed effects), the variation introduced by an individual population or mother individual was mostly relatively small (Tables 1 and 3; Figure 3).

**TABLE 3:** Standard deviation attributable to the random effects. Residual SDs are presented only for models where this parameter is well defined.

Response variable group	Response variable	SD (±CI) attributable to population	SD (±CI) attributable to mother ind.	SD (±CI) attributable to replicate	Residual SD
Leaf and flowering responses	Leaf size (√cm^2^)	0.1 (0.01, 0.3)	0.2 (0.04, 0.3)	0.3 (0.06, 0.7)	0.6 (0.6, 0.7)
	Number of leaves	0.06 (0.00, 0.1)	0.2 (0.1, 0.2)	0.2 (0.05, 0.6)	NA
	Flowering probability	0.6 (0.1, 1.2)	0.8 (0.3, 1.2)	0.4 (0.03, 1.2)	NA
	Number of flowers	0.2 (0.07, 0.4)	0.08 (0.0, 0.2)	0.3 (0.02, 0.9)	NA
Pigmentation responses	Total flavonoid content	27.8 (1.9, 65.2)	45.7 (6.3, 78.3)	19.8 (0.8, 63.7)	NA
	Total HCA content	25.7 (1.4, 62.1)	33.1 (2.2, 66.2)	17.2 (0.6, 58.3)	NA
	Blue light reflectance	0.02 (0.0, 0.06)	0.05 (0.0, 0.1)	0.05 (0.0, 0.2)	0.09 (0.06, 0.1)
Pathogen responses	Leaves with symptoms	0.08 (0.0, 0.2)	0.3 (0.2, 0.4)	0.4 (0.2, 1.1)	NA
	PlLV infections	0.3 (0.02, 0.8)	0.7 (0.1, 1.1)	0.7 (0.2, 1.5)	NA
	PLCaV infection	0.2 (0.01, 0.6)	0.4 (0.02, 1.0)	0.6 (0.03, 1.5)	NA
	Number of infections	0.1 (0.0, 0.4)	0.1 (0.01, 0.4)	0.4 (0.02, 1.1)	0.02 (0.0, 0.4)

For vegetative traits, blue light reflectance, individual virus infection and proportion of leaves with symptoms, the *mother individuals* introduced at least double the amount of variation compared with the *populations*. For flowering probability, total flavonoid, and HCA content, the *mother individuals* also introduced more variation than the *populations*, but the difference was smaller. For number of flowers, the pattern was the opposite with *population* introducing double the amount of variation of *mother individuals*. For number of infections, the *population* level and *mother individual* introduce the same amount of variation.

## 4 Discussion

### 4.1 Thermal Plasticity

Our study provides an overview of the thermal phenotypic plasticity in several traits, and how much this varies within and between populations of *P. lanceolata* at its high‐latitude range edge in the Åland Islands. Our results suggest that plants grown at the equivalent of their currently experienced mean or warmer temperatures have greater leaf size, flowering probability and abundance, more pathogen responses, higher flavonoid content, and greater blue light reflectance compared to those grown at colder temperatures. Overall, these findings are consistent with the general understanding that plants can exhibit flexibility in multiple traits to cope with variation in thermal environments (Ghalambor et al. 2007; Matesanz et al. 2010). However, the weak evidence for a difference in trait values between warm and mean temperatures suggests that their optimal temperature range may not have been fully captured in this experiment, and that some traits, such as flavonoid content and infections (especially by PlLV), peak at mean temperatures.

Interestingly, while most traits exhibited some degree of plasticity, especially when comparing the cold conditions to the two warmer ones, we found strong support for thermal plasticity in only a few traits. Notably, these were not consistently the traits that are considered to be less closely connected to fitness, such as leaf size, but instead high thermal plasticity was evident in traits more directly connected to fitness, such as flower production, pigmentation, and pathogen responses. These findings contrast with previous reports that traits critical to fitness tend to be more conserved and less plastic, likely due to the stronger selective pressures stabilizing these traits (Stearns and Kawecki 1994; Villellas et al. 2021). Previous studies have concluded that traits that confer less direct benefits to survival and reproduction would be afforded greater flexibility, allowing individuals to fine‐tune their responses to current environmental conditions. Notably, Villellas et al. (2021) tested the effect of different light and watering treatments on phenotypic plasticity in populations from across the range of *P. lanceolata* . They found that phenotypic plasticity tended to mask genetic differentiation for vegetative but not for reproductive traits, whereas genetic differentiation was apparent among reproductive traits. Although we were not able to specifically test genetic differentiation in this study, our results indicate that these patterns may be different for temperature compared to other environmental drivers such as drought. Plasticity patterns may also differ in this small portion of the species' geographical range from which the seeds were sampled.

Our correlation analysis revealed low correlations between measured traits, both across and within temperature treatments. This suggests that individual plants do not consistently exhibit high or low values across the suite of traits measured here. This points to trait independence, i.e., that trait plasticity is modulated independently of each other. Such trait independence could result from different genetic pathways or regulatory mechanisms being activated in response to temperature changes, which may in turn be driven by trade‐offs related to resource allocation.

### 4.2 Intrapopulational Versus Interpopulational Variation in Plasticity

Phenotypic plasticity has been suggested to be a critical mechanism for species in the face of rapid environmental changes (Matesanz et al. 2010; Sultan 1995). High intraspecific variation in such plasticity can allow populations to respond adaptively to climate change and to increasingly variable conditions (Walter et al. 2023). When focusing on the more thermally plastic traits in our study, we found that all but the flowering traits shared the pattern whereby variation in plasticity was mainly introduced from differences between mother individuals within the same population. However, our findings also indicate that the variation in plasticity stemming from both the intrapopulation and interpopulation levels was relatively minor in comparison to the differences resulting from different temperature conditions. This means that while populations are plastic to thermal conditions for many of the studied traits, and there is variation in this plasticity both between populations and between individuals stemming from the same mother, the average response norms *viz‐a‐viz* temperature are relatively similar across the studied populations. This underscores the risk that there may not be enough (epi)genetic or other individual or population‐level variation in the Åland *P. lanceolata* populations to provide material for rapid evolution to act upon. Nevertheless, there could be enough potential of populations to adapt to and persist under a changing climate, at least in the short term, if the recorded trait plasticity is adaptive when operating in combination with the observed higher intrapopulational variation compared to interpopulational (Cunningham et al. 2020). We did not compare trait values to fitness outcomes, so we can only speculate on the consequences of the recorded intrapopulational variation in plasticity. Although we found relatively high variation in trait values driven by thermal differences and replicates compared to intraspecific differences, the variation introduced by populations and individuals will not necessarily be negligible in its potential to affect population dynamics under a changing climate. The recorded larger variation in plasticity between individuals across Åland for vegetative traits, blue light reflectance, flavonoid content, PlLV infection, and pathogen symptoms that we found may be large enough to help sustain the specific population in a changing environment, as selection has been found to rapidly shift population responses under contemporary climate change (Franks et al. 2007).

This somewhat smaller role of interpopulation variation compared to intrapopulation variation could be indicative of a homogenizing effect of gene flow across this relatively small portion of the species' range. Microhabitat or other conditions at the site level may also vary more than the average variation between populations, thus supporting the persistence of intrapopulational variation. Maternal effects may also play a role in shaping trait expression. In this study, we did not attempt to remove maternal effects through a refresher generation; hence, parental effects reflecting conditions experienced by the mother individual could have affected the offspring's phenotypes in the thermal treatments (Lacey and Herr 2000). It is also possible that our overall results, based on the frequency of traits with higher intrapopulational variation, may be a spurious effect of the specific traits that we chose to measure, and had we have chosen others, we may have found another pattern.

The additional parameter that we included in some of our models allowed testing for differences in the variation of the response between different temperature treatments. It improved the fit for some of the traits tested, indicating that for leaf size, number of flowers and infections, and blue light reflectance, the variation in response differed in the different temperature treatments. Among these traits, we found some interesting patterns when compared to their overall plasticity and whether more variation was introduced from the intra‐ or interpopulational level. Leaf size and blue light reflectance showed high plasticity with more of the variation stemming from the intrapopulation level, with variation being highest in the warm and mean treatments, respectively. Number of flowers and infections were also thermally plastic but showed the highest variation in the cold. In addition, for number of flowers, the variation introduced was equal for the intra‐ and interpopulation levels, while little variation stemmed from either level for the number of infections. This highlights the importance of considering temperature‐specific differences in variation when studying thermal plasticity. It could also point to different adaptive potential being held within compared to between populations and that, in this case, more potential for optimal responses in warmer conditions could depend on intrapopulational variation. As this assumption is based on the results of only a few traits, we cannot draw definite conclusions regarding this; however, it offers an interesting study question for the future.

### 4.3 Pathogen and Pigmentation Responses

This is one of the first studies testing how temperature may affect the responses of *P. lanceolata* to seed‐borne viruses. We observed that the symptoms of pathogen infection that the plants displayed and the prevalence of PlLV infections were more pronounced in warmer conditions. However, the difference between thermal treatments in the number of infections was small, based on the two common viruses that we screened. This may suggest that only the symptoms, not the overall infection rates, were more severe in warmer conditions, or that the symptoms were caused by pathogens that we did not screen for in this study. The pathogens were introduced in the experiment via the seeds that we used—thus, this experiment does not mirror the same pathogen load that would be present in field conditions where pathogens may also be introduced from surrounding individuals and populations throughout the growing season. Nevertheless, while the pathogen responses that we measured allowed us to capture the outcome of seed‐borne pathogen effects in the different thermal treatments, we are not able to distinguish whether the plants differ in their resistance or whether the pathogens are more successful in the different temperatures (higher pathogen exposure). In addition, the symptoms that we observed could also have been caused by several other pathogens.

If our pathogen‐related observations are transferrable to a wider thermal range, this result might suggest that *P. lanceolata* will be more stressed in warmer conditions and thus could be more susceptible to pathogens under future warmer climates. It is, however, also possible that differences in pathogen exposure in different thermal settings can affect a host's ability to cope with thermal stress (Hector et al. 2021). The fact that we observed a two‐fold increase in PlLV infections between the cold and mean treatments indicates that climate change may indeed have an impact on subsequent vertical transmission of some of the virus species (Trebicki 2020). As both pathogen load and host resistance can have large effects on the dynamics of natural systems, the increase in pathogen responses with increased temperature that we detected presents an interesting hypothesis that warrants further study to shed light on its mechanistic underpinnings. The differences in displayed symptoms between the treatments were not major, and testing these dynamics under a larger range of temperatures could reveal whether pathogens and hosts have different optimal thermal ranges (Chen et al. 2024). Such studies could include prolonged periods of warm weather in combination with drought to elicit more realistic responses aligned with predicted future climatic conditions, highlighting the relative roles of pathogen sensitivity versus exposure.

Temperature treatment induced marked changes in the phenolic compound profile of *Plantago*. Specifically, total flavonoid content increased with higher temperatures, while the concentration of hydroxycinnamic acids (HCAs), including phenylethanoids, showed a decreasing trend, although this effect was not statistically significant. Such a pattern would be consistent with temperature‐induced activation of the flavonoid biosynthetic pathway, whereby HCA precursors could become depleted.

We also found increased blue‐light reflectance in bract tissues at elevated temperatures occurring concomitantly with these changes in flavonoid and HCA accumulation. This suggests that anthocyanin also declined under these conditions. This pattern would align with previous findings that anthocyanin biosynthesis is typically upregulated at lower temperatures in various plant species, including *P. lanceolata* (Stiles et al. 2007). Given that flavonoids serve as substrates for anthocyanin biosynthesis, downregulation of anthocyanin accumulation at higher temperatures may also contribute to the accumulation of intermediate flavonoid compounds.

The biological implications of these temperature‐driven metabolic shifts are potentially diverse. Changes in bract optical properties—particularly the ratio of UV‐A to blue light reflectance—may alter visual cues used by pollinators, as the spectral contrast is influenced by the relative abundance of flavonoids and anthocyanins (Narbona et al. 2021). Moreover, we speculate that the temperature‐dependent modulation of phenolic metabolism could also underlie observed variations in pathogen responses. A decline in HCA concentration may weaken chemical defenses, as numerous HCA derivatives are known to have antimicrobial, antiviral, and antifungal activities. Among these, Plantago phenylethanoids such as verbascoside (acteoside) and various caffeic acid derivatives have antiviral properties against human and animal viruses. While effects are mostly mediated through immunomodulatory pathways, direct inhibition of viral DNA replication has also been reported (Chathuranga et al. 2019). However, it remains to be determined whether such compounds contribute directly to resistance against plant viral pathogens, as empirical evidence in this context is currently lacking.

## 5 Conclusions and Future Directions

Our study underscores the importance of studying pathogen responses under changing climatic regimes, as we found indications for increased frequency of pathogen symptoms in warmer temperatures and since the interaction between pathogen exposure and sensitivity can have far‐reaching consequences on fitness. This is particularly important for species at their range margins, where the pressures of climate change may be highest and thus the need for adaptive capacity is the greatest. Our study further highlights the interplay between overall thermal plasticity in multiple traits and how it varies over a hierarchy of individual and population‐level plasticity. Together, these differences in plasticity will shape population‐level responses to fluctuating thermal conditions and may vary substantially between traits.

Overall, our results suggest that *P. lanceolata* in Åland will probably thrive under warmer thermal conditions, at least within the bounds of the temperatures used in this experiment and assuming sufficient moisture. Precipitation extremes, because of increasing variability in the weather, are in many places a more likely consequence of climate change than temperature extremes (van der Wiel and Bintanja 2021). Indeed, drought likely has a larger effect on this species compared to temperature (van Bergen et al. 2020; Villellas et al. 2021). Future experiments imposing temperature manipulations on *P. lanceolata* should not only apply larger increments between temperature treatments to identify the optimum and approach the limits of thermal plasticity, but also use more complicated factorial designs including interactions with multiple environmental factors, such as a combination of light, drought, and temperature to assess the extent of thermal plasticity in interaction with other environmental factors. It would also be important to specifically assess the genetic component of plasticity.

Considering thermal effects on *P. lanceolata* , which was the focus of this study, the substantially smaller role of intraspecific variation in plasticity compared to the effects of temperature that we found could mean that, under abrupt changes in thermal conditions, all populations within the study region would be similarly affected. Such similarity in thermal response among populations potentially leaves them susceptible to declines if thermal extremes surpass their tolerance limits. This is accentuated by the findings of recent studies in the study region, indicating that spatial variation in weather conditions has decreased and extremes have increased (Kahilainen et al. 2018). Such changes lead to an increased likelihood that similar weather conditions will prevail over the entire study area simultaneously. However, here, the slightly greater individual‐level variation in most of the thermally plastic traits that we found may provide a crucial buffer, allowing for rapid population recovery and adaptation following severe climatic events. Given this finding, we conclude that the potential for evolving novel plastic responses and providing a source of resilience under climatic fluctuations is more likely harbored within the Åland *P. lanceolata* populations than between them. If such plasticity is adaptive and also promotes the evolution of novel responses (cf. Oostra et al. 2018), measuring it can help assess the capacity of populations to adjust to novel and potentially more variable conditions both in current locations at the range edge and in newly colonized areas beyond the range front. The implications of our findings extend to the management of the ecological system underpinned by *P. lanceolata* (Opedal et al. 2020). The indications that many thermally plastic traits, including vegetative, pigmentation, and pathogen responses, exhibit higher intrapopulational variability in thermal plasticity, suggest that management strategies should support not only large enough habitats and population sizes but also microhabitat diversity within populations. The latter could be promoted by ensuring a suitable grazing regime of pastures to support these multispecies communities (Li et al. 2021). Such efforts can help maintain the genetic and phenotypic diversity necessary for adaptive responses in this plant species and ensure that it can continue to thrive and provide benefits to other species that depend on it.

## Author Contributions

**M. H. Hällfors:** conceptualization (lead), data curation (lead), formal analysis (lead), funding acquisition (lead), investigation (lead), methodology (lead), project administration (lead), resources (lead), supervision (lead), validation (equal), visualization (lead), writing – original draft (lead), writing – review and editing (lead). **T. M. Robson:** conceptualization (supporting), investigation (supporting), methodology (equal), project administration (equal), resources (equal), supervision (supporting), validation (equal), writing – original draft (equal), writing – review and editing (equal). **S. Burg:** conceptualization (supporting), data curation (supporting), investigation (supporting), methodology (supporting), project administration (supporting), writing – review and editing (supporting). **S. Pentikäinen:** data curation (supporting), investigation (supporting), methodology (supporting), project administration (supporting), writing – review and editing (supporting). **S. H. M. Koivusaari:** formal analysis (supporting), investigation (supporting), validation (supporting), visualization (equal), writing – review and editing (supporting). **M. Luoto:** conceptualization (supporting), methodology (supporting), resources (supporting), validation (supporting), writing – review and editing (supporting). **J. Nezval:** data curation (supporting), investigation (supporting), methodology (supporting), resources (equal), validation (supporting), visualization (supporting), writing – original draft (supporting), writing – review and editing (equal). **R. Pech:** data curation (supporting), investigation (supporting), methodology (supporting), resources (supporting), validation (supporting), writing – original draft (supporting), writing – review and editing (supporting). **M. Saastamoinen:** conceptualization (supporting), funding acquisition (supporting), methodology (supporting), resources (supporting), validation (supporting), writing – review and editing (equal). **L. E. Schulman:** investigation (supporting), resources (supporting), validation (supporting), writing – review and editing (supporting). **J. Sirén:** formal analysis (equal), methodology (supporting), validation (equal), writing – original draft (supporting), writing – review and editing (supporting). **H. Susi:** conceptualization (supporting), data curation (supporting), investigation (supporting), methodology (supporting), resources (equal), supervision (supporting), validation (equal), writing – original draft (supporting), writing – review and editing (equal).

## Conflicts of Interest

The authors declare no conflicts of interest.

## Supporting information


**Data S1:** ece372201‐sup‐0001‐supinfo.docx.

## Data Availability

Data and code for reproducing the results are available in Dryad (https://doi.org/10.5061/dryad.pvmcvdnxp). Figures S1–S7 and Tables S1–S3 are available in the “Supporting Information.docx” as part of the accompanying data in Dryad.
