# Supplementary material for: Thermal Plasticity of Multiple Traits Varies More Within Than Between Populations of Plantago lanceolata at Its Northern Range Edge
Source: Ecol Evol. 2025 Oct 21;15(10):e72201. doi: 10.1002/ece3.72201 (PMC12539371; doi:10.1002/ece3.72201)
Supplement: Supplementary file 1 — Data S1: ece372201‐sup‐0001‐supinfo.docx. [file ECE3-15-e72201-s001.docx]

**Supplementary Information for:**

Hällfors, M.H., Robson T.M., Burg S., Pentikäinen S., Koivusaari S. H. M., Luoto M., Nezval J., Pech R., Saastamoinen M., Schulman L., Sirén J., Susi H.: Thermal plasticity of multiple traits varies more within than between populations of *Plantago lanceolata* at its northern range edge. **Contents:**
Figures S1-S7
Table S1-S3
References


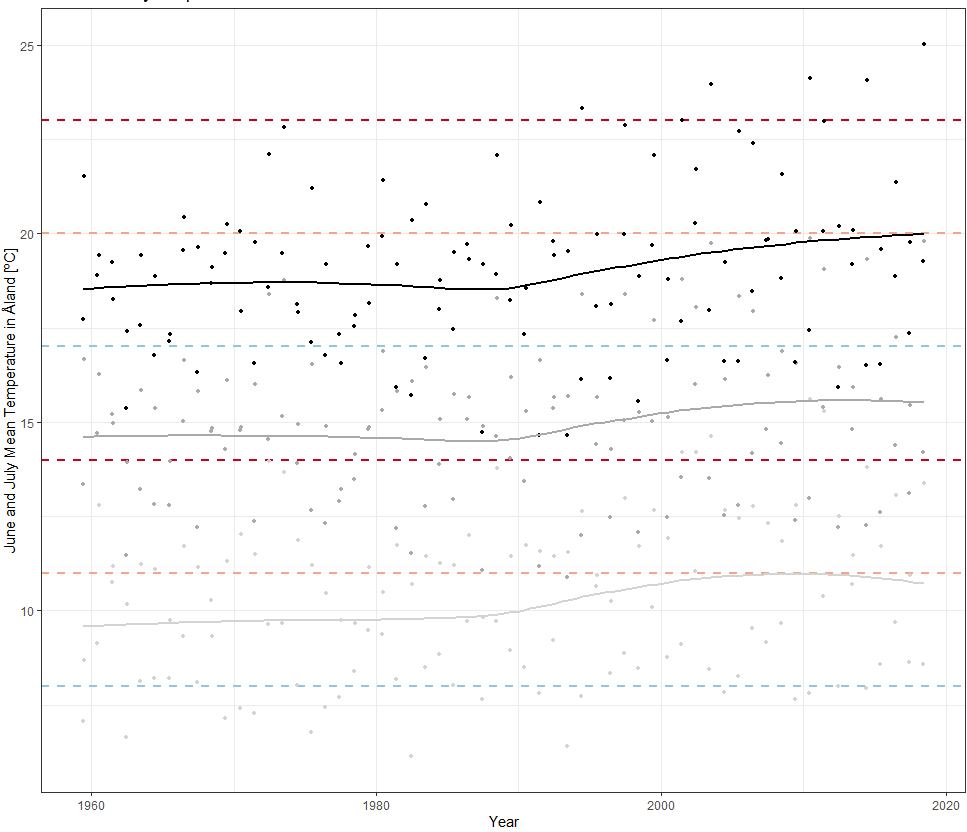


**Figure S1. June and July temperature in Åland, Finland during 1960-2020 in relation to experimental treatments.** The point and lines in light grey, grey and dark grey show minimum, median and maximum temperatures, respectively. The lines are smoothed conditional means fitted using *ggplot* in R (Wickham, 2016). The dashed horizontal lines in blue, peach and red show night (lower) and day (upper) temperatures set for the thermal experiments for the cold, mean and warm treatments, respectively.


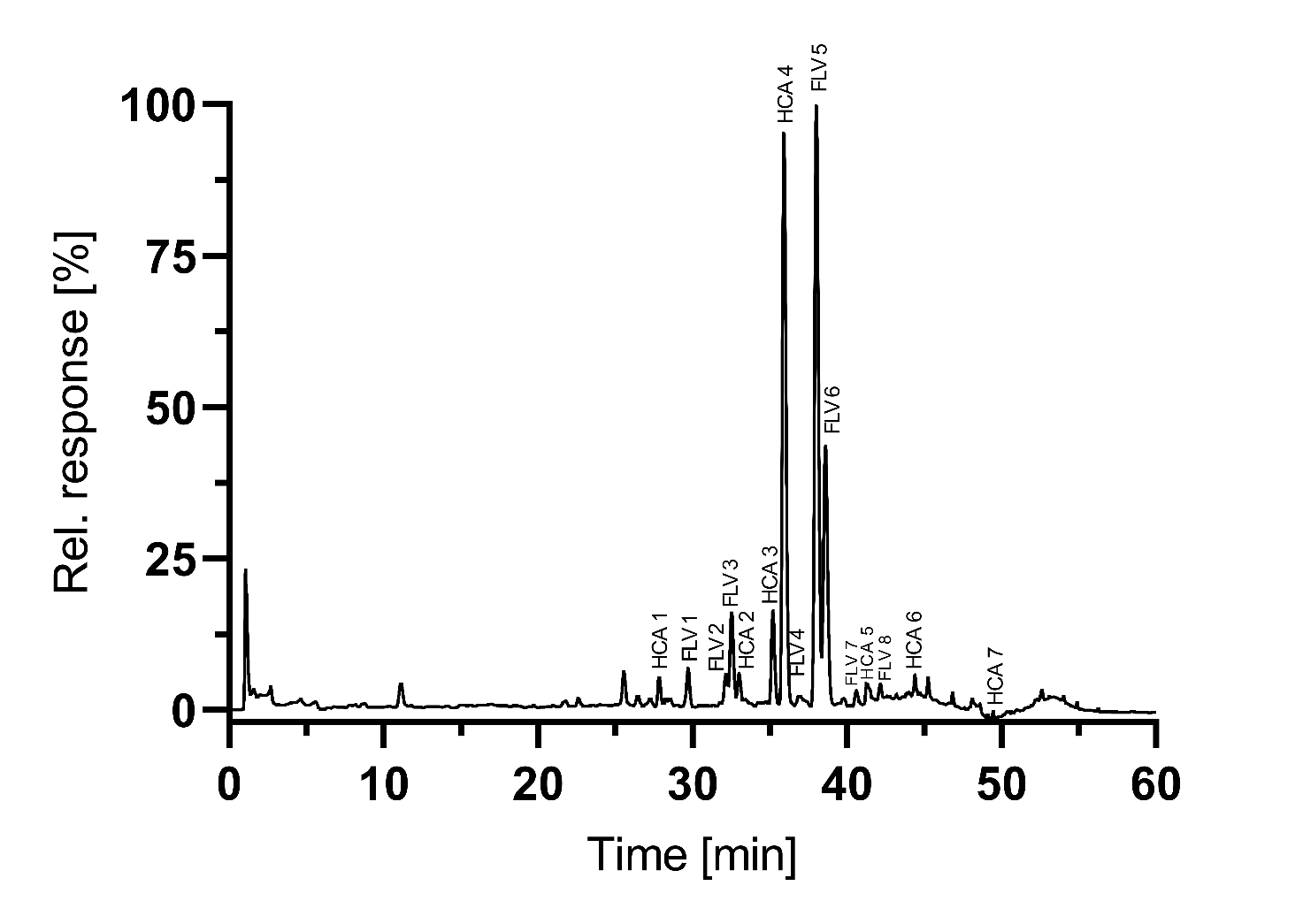


**Figure S2.** Typical chromatogram of separated phenolic compounds obtained from HPLC-DAD analysis recorded at 270 nm. HCA – hydroxycinnamic acid derivatives; FLV – flavonoids.


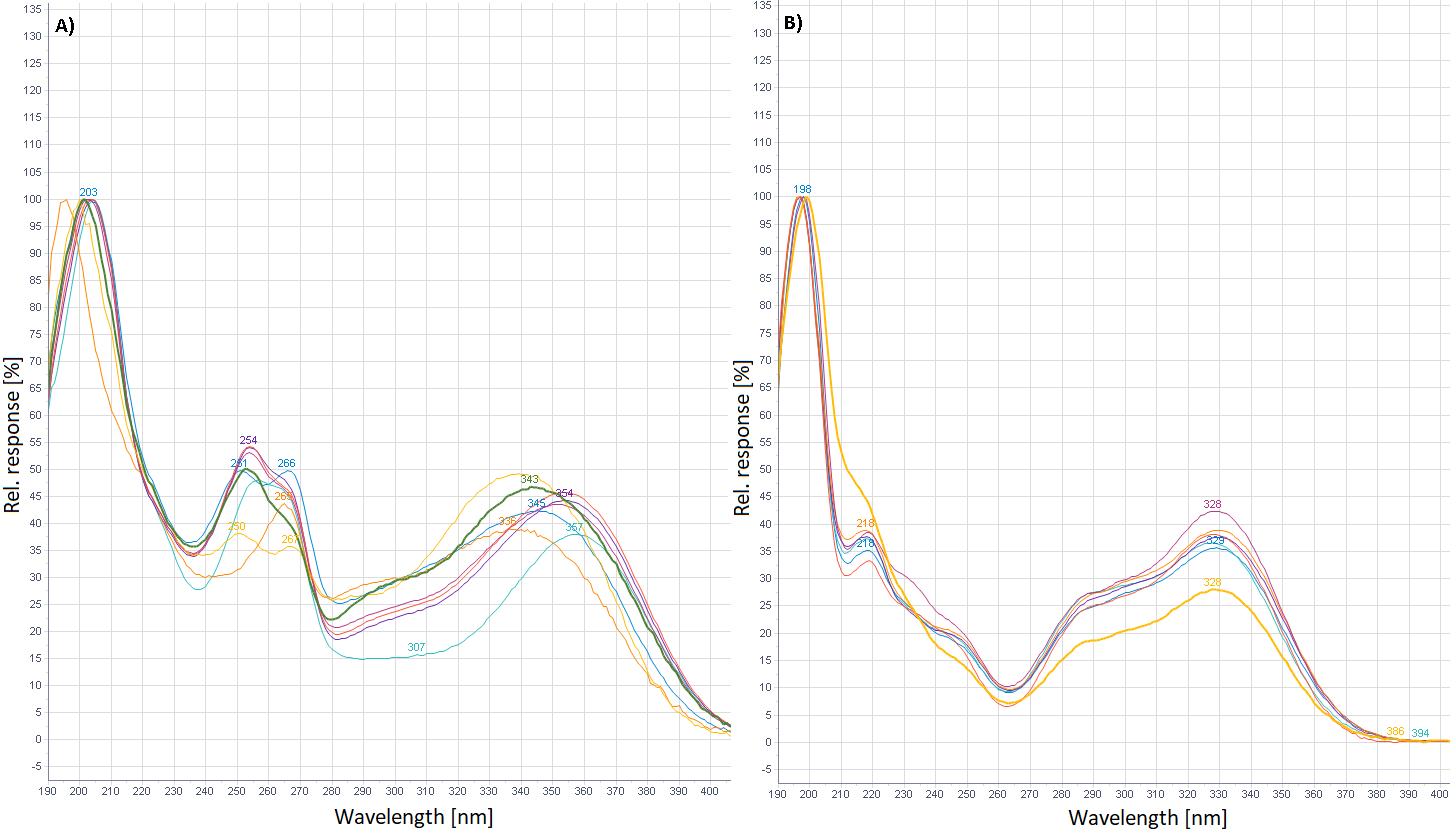


**Figure S3.** UV-VIS absorption spectra of phenolic compounds detected by HPLC-DAD**.** A) flavonoid derivatives, B) hydroxycinnamic acid derivatives.


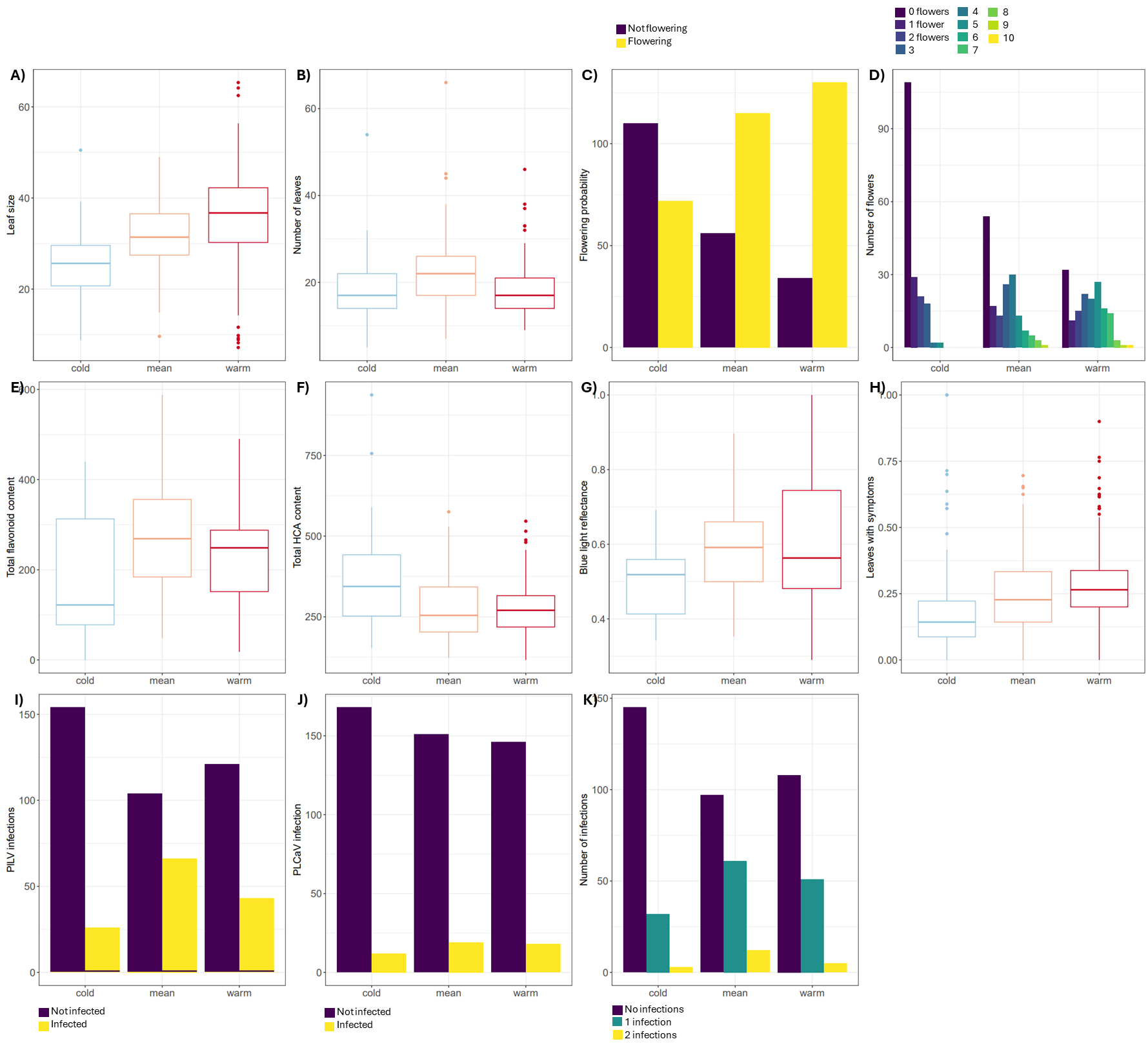


**Fig S4.** **Measured trait response across temperature treatment** (summarized across populations) for A) leaf size, B) number of leaves; C) flower presence; D) number of flowers; E) flavonoid content of peak area mAU.s/ d.w. mg; F) HCA content; G) blue light reflection of inflorescence bracts; H) proportion of leaves with pathogen symptoms; I) number of individuals with or without PLCaV infection J) number of individuals with or without PlLV infection; K) total number virus infections per individual (0, 1=either PLCaV or PlLV infection; 2=both infections). Data presented per population in Fig S4. Estimated effects in main text Fig. 2 and Table 1.


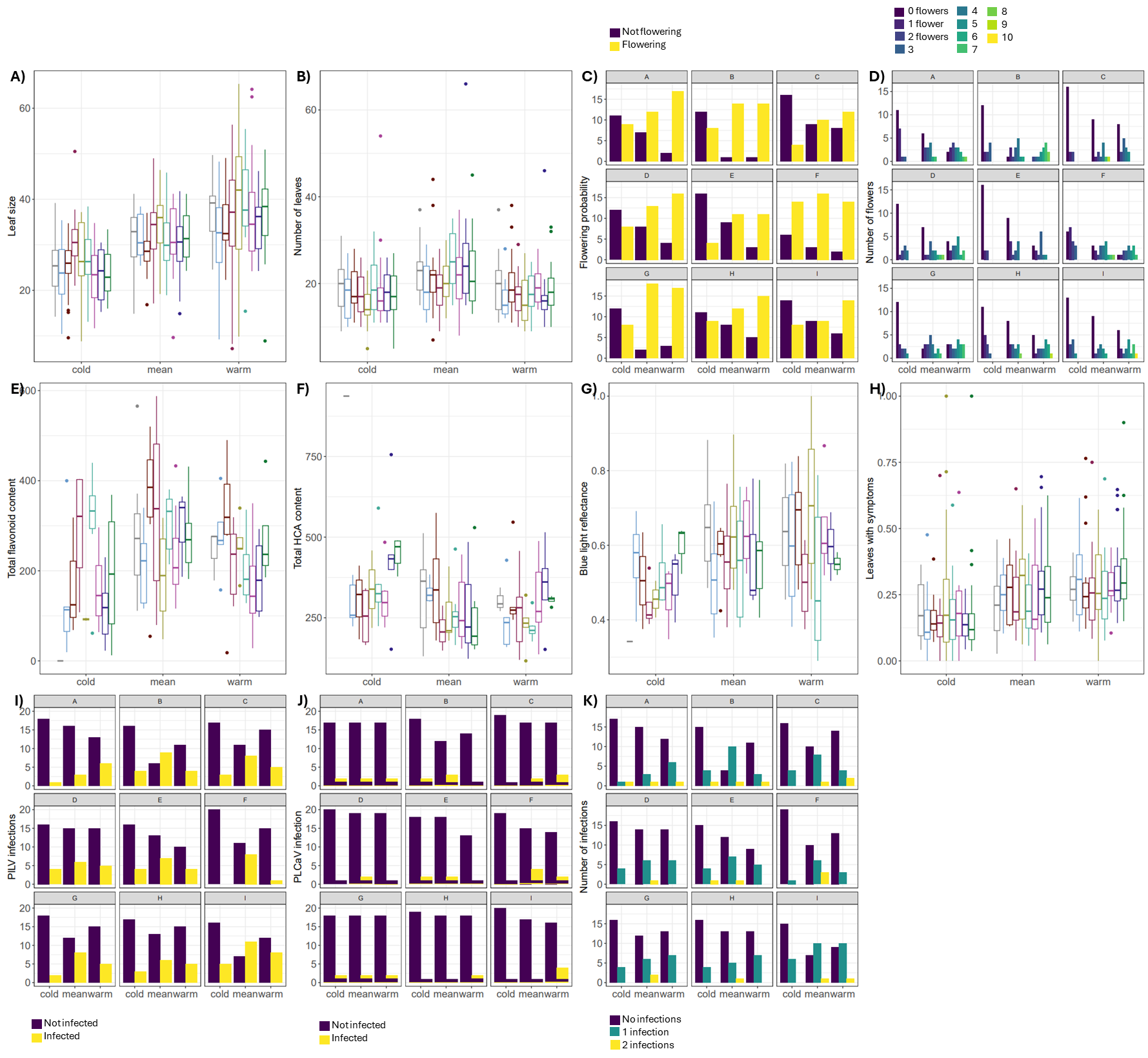


**Fig S5.** **Measured trait response across temperature treatment** (separately per population) for A) leaf size, B) number of leaves; C) flower presence; D) number of flowers; E) flavonoid content of peak area mAU.s/ d.w. mg; F) HCA content; G) blue light reflection of inflorescence bracts; H) proportion of leaves with pathogen symptoms; I) number of individuals with or without PLCaV infection J) number of individuals with or without PlLV infection; K) total number virus infections per individual (0, 1=either PLCaV or PlLV infection; 2=both infections). Data presented per across populations in Fig S3. Estimated effects per population in Fig. S7.


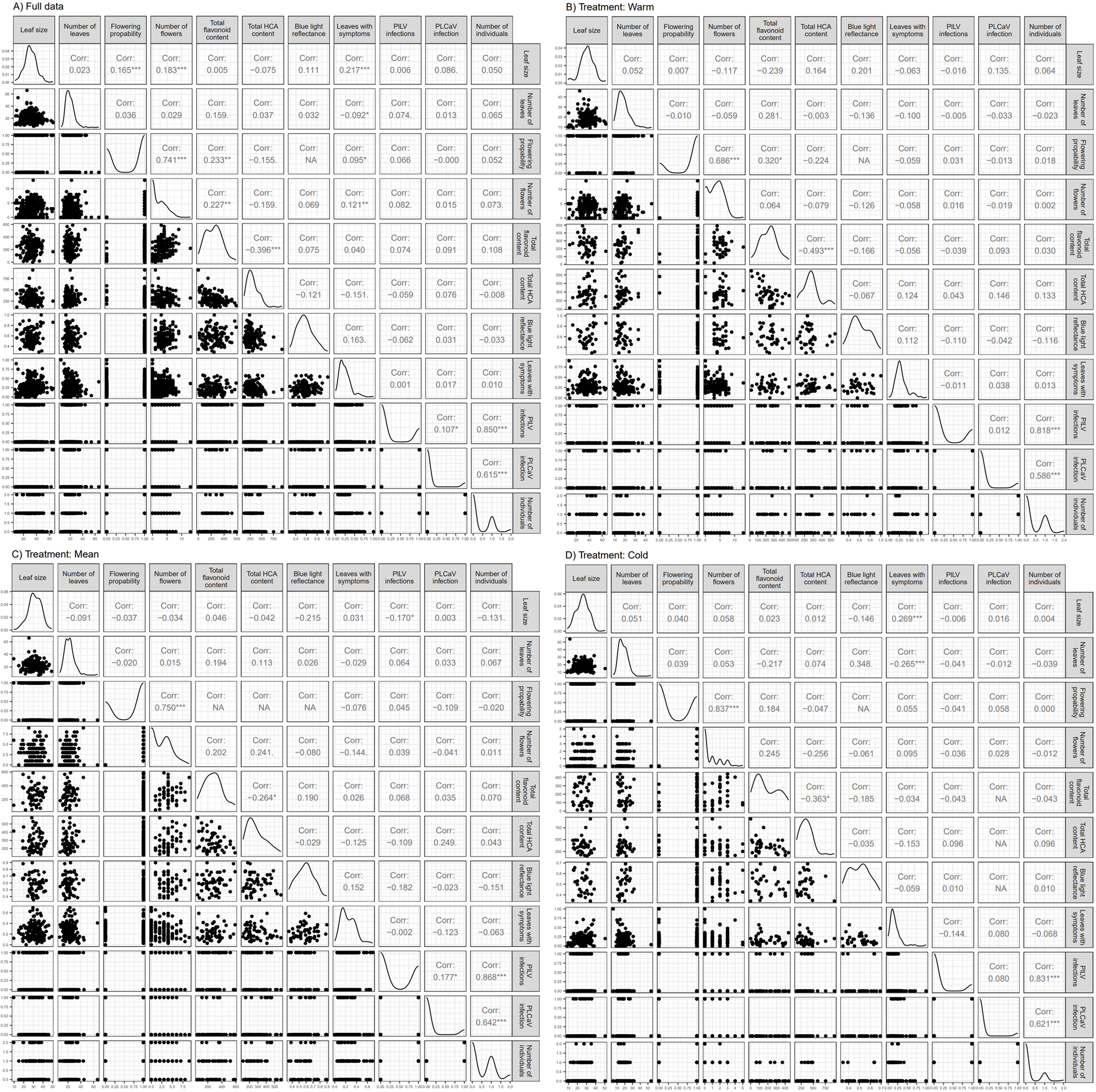


**Figure S6. Correlation plots of measured traits** A) across treatments, and in the B) cold, C) mean and D) warm treatments. The correlation plots were created using the ggpairs function in the GGally pacakage in R (Schloerke et al., 2024).


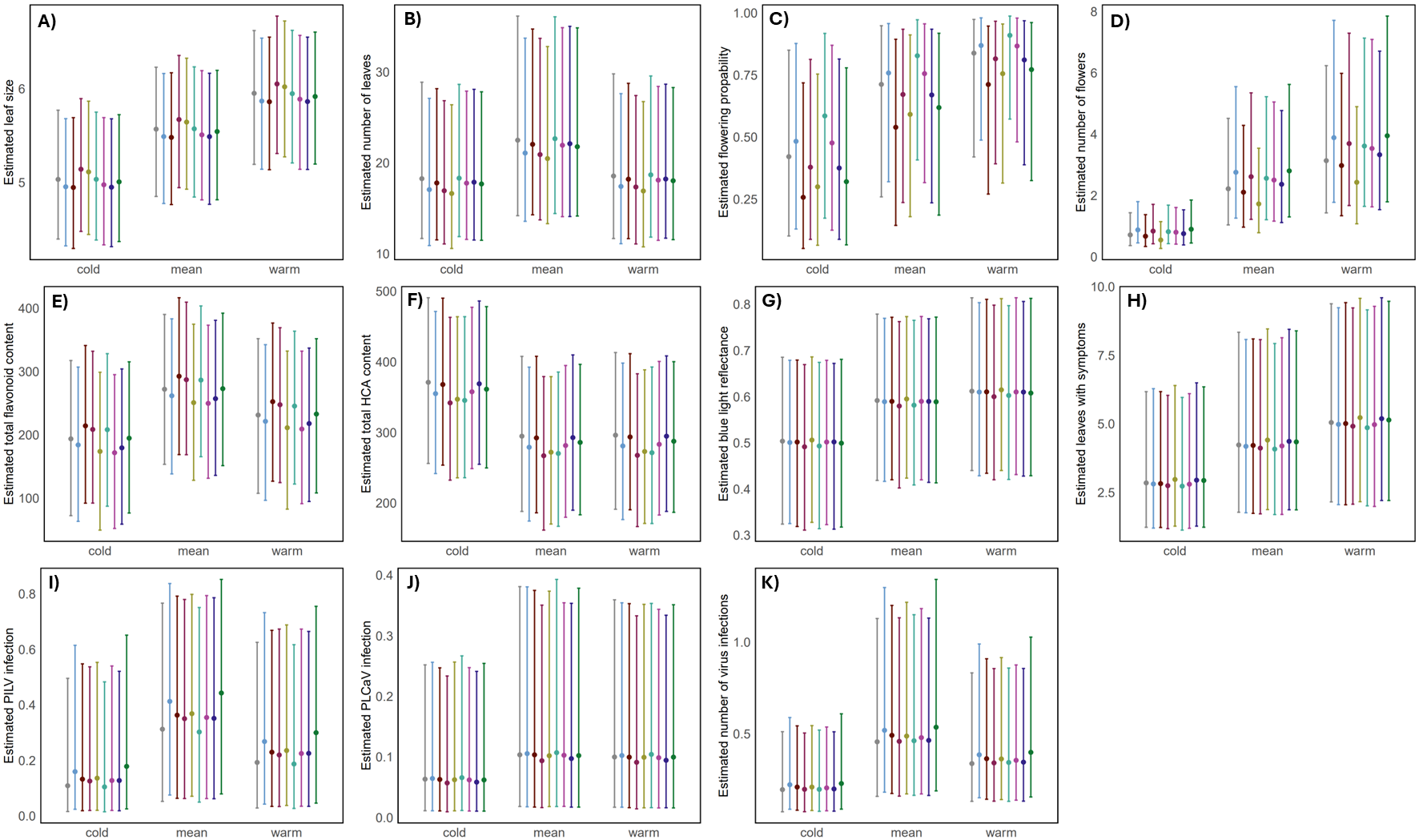


**Fig S7. Posterior mean and credible intervals for the average response per treatment temperature and population (different colors).** A) Leaf size, B) number of leaves; C) flowering probability; d) number of flowers, E) flavonoid content; F) HCA content; G) blue light reflection; H) proportion of leaves with pathogen symptoms; I) probability of PLCaV infection J) probability of PlLV infection; K) probability of virus infections. Estimated effects (on the latent, i.e., modelled, scale) in main text Table 1. Estimated effects across populations in main text Fig 2.

**Table S1. Information on seed sampling locations.**

| **Population** | **Sampled individuals** | **Y-coord** | **X-coord** | **Location** | **Municipality** |
| --- | --- | --- | --- | --- | --- |
| A | 50 | 60.06770 | 20.071867 | Söderby | Lemland |
| B | 50 | 60.13275 | 19.989163 | Österkalmare | Jomala |
| C | 50 | 60.17423 | 19.523903 | Skeppsvik | Eckerö |
| D | 28 | 60.22434 | 19.559542 | Storby | Eckerö |
| E | 17 | 60.27522 | 20.240213 | Hulta | Sund |
| F | 39 | 60.25415 | 20.228615 | Mångstekta | Sund |
| G | 26 | 60.26042 | 20.178783 | Strömbolstad | Sund |
| H | 20 | 60.08355 | 19.906516 | Gregersö, Möckelö | Jomala |
| I | 48 | 60.06605 | 19.951663 | Espholm, Ytternäs | Mariehamn |

**Table S2. Summary of model statistics for leaf size and inflorescence presence for models where one of the highly connected populations (E, F, G) was removed at a time.** Part a) Mean of the posterior probability distribution with lower and upper credible intervals. Estimates are on the linear predictor scale. Part b) evidence ratio for a one-sided hypothesis asking what the posterior probability is of the response being bigger than 0 in the mean temperature, between the mean and the warm temperature, and in the warm temperature.

| **Response variable / sensitivity model** | **Estimate in cold (±CI)** | **Estimate in mean (±CI)** | **Estimate in warm (±CI)** | **Post. prob. of the resp. in the mean > 0** | **Post. Prob. of the resp. in the warm > 0** | **Post. Prob. of the diff. btw warm and mean > 0** |
| --- | --- | --- | --- | --- | --- | --- |
| Leaf size (√cm^2^) | 5.0 (4.6, 5.5) | 5.5 (5.1, 6.0) | 5.9 (5.4, 6.4) | **0.96** | **0.99** | 0.93 |
| Leaf size (√cm^2^) E pop. Removed | 5.01 (4.59, 5.51) | 5.52 (5.04, 5.95) | 5.91 (5.41, 6.36) | **0.96** | **0.99** | 0.93 |
| Leaf size (√cm^2^) F pop. Removed | 5.01 (4.59, 5.51) | 5.51 (5.04, 5.95) | 5.91 (5.41, 6.36) | **0.96** | **0.99** | 0.93 |
| Leaf size (√cm^2^) G pop. Removed | 5.05 (4.64, 5.44) | 5.56 (5.15, 5.95) | 5.92 (5.48, 6.32) | **0.97** | **0.99** | 0.93 |
| Flowering probability | -0.4 (-1.3, 0.6) | 0.8 (-0.1, 1.7) | 1.6 (0.6, 2.5) | **0.97** | **0.99** | 0.93 |
| Flowering probability E pop. removed | -0.30 (-1.15, 0.69) | 0.87 (-0.04, 1.75) | 1.55 (0.61, 2.45) | **0.97** | **0.99** | 0.92 |
| Flowering probability F pop. removed | -0.60 (-1.37, 0.34) | 0.8 (-0.23, 1.54) | 1.44 (0.48, 2.31) | **0.97** | **0.99** | 0.93 |
| Flowering probability G pop. removed | .0.42 (-1.33, 0.63) | 0.66 (-0.32, 1.62) | 1.52 (0.46, 2.47) | **0.96** | **0.99** | 0.94 |

**Table S3. Standard deviation attributable to the random effects for models on leaf size and inflorescence presence where one of the highly connected populations (E, F, G) was removed at a time.** Residual SDs are presented only for the leaf size model where this parameter is well defined.

| **Response variable / sensitivity model** | **SD (±CI) attributable to *population*** | **SD (±CI) attributable to *mother ind.*** | **SD (±CI) attributable to *replicate*** | **Residual standard deviation** |
| --- | --- | --- | --- | --- |
| Leaf size (√cm^2^) | 0.1 (0.01,0.3) | 0.2 (0.04,0.3) | 0.3 (0.06,0.7) | 0.6 (0.6,0.7) |
| Leaf size (√cm^2^) E pop. removed | 0.11 (0.01,0.28) | 0.19 (0.04,0.30) | 0.25 (0.06,0.75) | 0.62 (0.55,0.71) |
| Leaf size (√cm^2^) F pop. removed | 0.11 (0.01,0.28) | 0.19 (0.04,0.30) | 0.25 (0.06,0.75) | 0.62 (0.55, 0.71) |
| Leaf size (√cm^2^) G pop. removed | 0.14 (0.01,0.33) | 0.19 (0.04,0.30) | 0.22 (0.04,0.66) | 0.63 (0.56,0.72) |
| Flowering probability | 0.6 (0.1,1.2) | 0.8 (0.3,1.2) | 0.4 (0.03,1.2) | NA |
| Flowering probability E pop. removed | 0.61 (0.14,1.21) | 0.73 (0.22,1.17) | 0.38 (0.02,1.15) | NA |
| Flowering probability F pop. removed | 0.43 (0.04,1.01) | 0.77 (0.33,1.19) | 0.39 (0.02,1.19) | NA |
| Flowering probability G pop. removed | 0.62 (0.15,1.25) | 0.85 (0.41,1.30) | 0.45 (0.03,1.25) | NA |

**References**

Anderson, E. R., Lovin, M. E., Richter, S. J., & Lacey, E. P. (2013). Multiple Plantago species (Plantaginaceae) modify floral reflectance and color in response to thermal change. *American Journal of Botany*, *100*(12), 2485–2493. https://doi.org/10.3732/ajb.1300180

Guo, X., Hao, X., Zheng, J. M., Little, C., & Khosla, S. (2016). Response of greenhouse mini-cucumber to different vertical spectra of LED lighting under overhead high pressure sodium and plasma lighting. *Acta Horticulturae*, *1134*, 87–94. https://doi.org/10.17660/ActaHortic.2016.1134.12

Jewell, J., McKEE, J., & Richards, A. J. (1994). The keel colour polymorphism in Lotus corniculatus L.: Differences in internal flower temperatures. *New Phytologist*, *128*(2), 363–368. https://doi.org/10.1111/j.1469-8137.1994.tb04020.x

Lacey, E. P., & Herr, D. (2005). Phenotypic plasticity, parental effects, and parental care in plants? I. An examination of spike reflectance in Plantago lanceolata (Plantaginaceae). *American Journal of Botany*, *92*(6), 920–930. https://doi.org/10.3732/ajb.92.6.920

Mckee, J., & Richards, A. J. (1998). Effect of flower structure and flower colour on intrafloral warming and pollen germination and pollen-tube growth in winter flowering Crocus L. (Iridaceae). *Botanical Journal of the Linnean Society*, *128*(4), 369–384. https://doi.org/10.1111/j.1095-8339.1998.tb02127.x

Norberg, A., Susi, H., Sallinen, S., Baran, P., Clark, N. J., & Laine, A.-L. (2023). Direct and indirect viral associations predict coexistence in wild plant virus communities. *Current Biology*, *33*(9), 1665-1676.e4. https://doi.org/10.1016/j.cub.2023.03.022

Sallinen, S., Norberg, A., Susi, H., & Laine, A.-L. (2020). Intraspecific host variation plays a key role in virus community assembly. *Nature Communications*, *11*(1), 5610. https://doi.org/10.1038/s41467-020-19273-z

Sallinen, S., Susi, H., Halliday, F., & Laine, A.-L. (2023). Altered within- and between-host transmission under coinfection underpin parasite co-occurrence patterns in the wild. *Evolutionary Ecology*, *37*(1), 131–151. https://doi.org/10.1007/s10682-022-10182-9

Schloerke, B., Cook, D., Larmarange, J., Briatte, F., Marbach, M., Thoen, E., Elberg, A., Toomet, O., Crowley, J., Hofmann, H., & Wickham, H. (2024). *GGally: Extension to “ggplot2”* (Version 2.2.1). https://cran.r-project.org/web/packages/GGally/index.html

Stiles, E. A., Cech, N. B., Dee, S. M., & Lacey, E. P. (2007). Temperature-sensitive anthocyanin production in flowers of Plantago lanceolata. *Physiologia Plantarum*, *129*(4), 756–765. https://doi.org/10.1111/j.1399-3054.2007.00855.x

Susi, H., Filloux, D., Frilander, M. J., Roumagnac, P., & Laine, A.-L. (2019). Diverse and variable virus communities in wild plant populations revealed by metagenomic tools. *PeerJ*, *7*, e6140. https://doi.org/10.7717/peerj.6140

Susi, H., & Laine, A.-L. (2015). The effectiveness and costs of pathogen resistance strategies in a perennial plant. *Journal of Ecology*, *103*(2), 303–315. https://doi.org/10.1111/1365-2745.12373

Trebicki, P. (2020). Climate change and plant virus epidemiology. *Virus Research*, *286*, 198059. https://doi.org/10.1016/j.virusres.2020.198059

Villellas, J., Ehrlén, J., Crone, E. E., Csergő, A. M., Garcia, M. B., Laine, A.-L., Roach, D. A., Salguero-Gómez, R., Wardle, G. M., Childs, D. Z., Elderd, B. D., Finn, A., Munné-Bosch, S., Bachelot, B., Bódis, J., Bucharova, A., Caruso, C. M., Catford, J. A., Coghill, M., … Buckley, Y. M. (2021). Phenotypic plasticity masks range-wide genetic differentiation for vegetative but not reproductive traits in a short-lived plant. *Ecology Letters*, *24*(11), 2378–2393. https://doi.org/10.1111/ele.13858

Wickham, H. (2016). *ggplot2: Elegant Graphics for Data Analysis*. Springer-Verlag New York. https://ggplot2.tidyverse.org
